# Bridging the knowledge gap: a feminist lens on the post-birth control syndrome and media narratives

**DOI:** 10.3389/fgwh.2025.1556810

**Published:** 2025-07-09

**Authors:** Jana Niemann, Amand Führer

**Affiliations:** ^1^ Institute of Medical Sociology, Interdisciplinary Center of Health Sciences, Medical Faculty, Martin Luther University Halle-Wittenberg, Halle, Germany; ^2^ Institute for Medical Epidemiology, Biometrics and Informatics, Martin Luther University Halle-Wittenberg, Halle, Germany

**Keywords:** post-birth control syndrome, oral contraception, medicalization, discontinuation, contraceptive pill, online communities

## Abstract

**Background:**

Research regarding the period after the discontinuation of oral contraceptives remains largely confined to the return of fertility and menstruation, reflecting a narrow and medicalizing approach to sexual and reproductive health. Still, beyond this biomedical discourse, there is a growing debate concerning the experiences of those who discontinue oral contraception. This debate gravitates around the term “post-birth control syndrome” and mostly takes place in various online communities.

**Aim:**

In this perspective, we aim to explore this discourse on German and English language (social) media and highlight how the absence of reliable studies creates a void that is actively filled by commercial agendas, unchecked online information, and personal accounts.

**Argument:**

Instead of merely turning to the internet because of insufficient research, it is the commercial exploitation of this gap that intensifies certain narratives, risking spreading misinformation and disempowering former pill users by depriving them of scientific perspectives on their health. We emphasize the necessity of addressing this gap through feminist research that prioritizes biopsychosocial well-being, including its structural dimensions, rather than focusing solely on reproductive outcomes. Bridging this knowledge gap requires classical clinical and socioepidemiological research into post-pill physiological mechanisms and contexts, complemented by qualitative studies capturing user experiences.

**Conclusion:**

By shifting contraceptive health research towards comprehensive, user-centered perspectives, feminist science can empower individuals to make informed decisions and promote contraceptive autonomy within medical and public health frameworks.

## 1 Introduction

The internet and social media can provide spaces for connection and exchange on health issues. However, they might as well spread misinformation (1–3), especially when there is little to no research on a specific health issue, and medical claims spread on the internet cannot be fact-checked against clinical studies.

For decades, feminist scholars have pointed out the neglect of research regarding women's^1^ health topics and gender-sensitive medicine (4–6). Hereby, the discontinuation of oral contraceptives (OCPs) is a case in point: There is only limited research on that topic, and as of now, there are only a few studies that take the lived experience of people who experience unwanted effects after discontinuation into account (7–9). Instead, most research on what occurs after OCP discontinuation is limited to the return of fertility and menstruation (10, 11). From a feminist perspective^2^, this situation is problematic since it restricts research on the narrow topic of womxn's reproductive responsibility and ignores their overall biopsychosocial health and the need for alternative contraceptive options following discontinuation (8, 12, 13). It also expresses a dismissive attitude towards the post-discontinuation experiences of former pill users, while simultaneously shining a light on the androcentric tendencies prevalent in the medical field (14).

At the same time, this gap has been filled in online discourse with references to the “post-birth control syndrome” (PBCS), which introduces another set of problems: While PBCS is not recognized as a reality among gynecologists and the wider medical community, it is an area of growing public interest and concern. Since health science fails to provide input into this discourse, PBCS is a good example of what can happen – positive and negative – when science implicitly declares itself “not responsible”.

Therefore, in this perspective, we aim to provide a short illustrative summary of this online discourse, show its problems, and outline future research directions regarding PBCS.

## 2 Information about the (dis)continuation of the contraceptive pill

Access to reliable information is one important component of sexual and reproductive agency (15). This also applies to information on contraceptives. However, since the introduction of OCPs, the debate about potential adverse effects, their impact on the broader physiology of hormones, and the issue of discontinuation has been contentious. This ongoing debate in the scientific community and (social) media may have impacted how women reflect on the decision to (dis)continue OCPs (2, 3, 16).

In fact, studies indicate that the frequency of OCP use in Western Europe has declined in recent decades (17–20). Factors influencing OCP discontinuation include the experience of adverse effects (21–23), changes in the risk perception of adverse effects (24), communication with peers and healthcare providers (22, 24), desire for natural contraception (9, 21), and social media influence (2, 16). Social and mainstream media play a key role in the search for contraceptive information, especially when research has found a disconnect between contraceptive counselors and (potential) users (22, 25, 26).

## 3 Post-birth control syndrome

Therefore, a growing number of studies started looking into the characteristics of birth control-related online information. Still, this ever-increasing field of inquiry has substantial gaps: While working on a paper regarding YouTube and the contraceptive pill (9), the first author came across the PBCS. This term originated from Romm's book “Botanical Medicine for Women's Health” in 2008. Per Romm, the term encompasses the irregular menstrual cycles and other related symptoms that certain individuals may encounter for several months following the cessation of hormonal contraceptive use. Furthermore, Joana Brighten defined the PBCS as “*a constellation of symptoms women experience, when they discontinue hormonal birth control*” in her book “Beyond the Pill: A 30-Day Program to Balance Your Hormones, Reclaim Your Body, and Reverse the Dangerous Side Effects of the Birth Control Pill.” (27). Brighten states the 'syndrome' occurs three to six months after discontinuation. However, she also used the book and her website as a pitch for her line of nutritional supplements and her naturopathic program, claiming that they, for instance “*harmonize ovarian hormones*” and “*reverse metabolic mayhem*” (28).

The widespread appeal of these stories and products highlights a broader trend in which people dealing with post-pill symptoms seek commercial and alternative options, owing to a lack of accessible and reliable medical advice. In this context, one might suggest to “consult your doctor”, when seeking online information and taking supplements. This often overlooks the fact that not all former users have reliable and consistent access to healthcare. This is especially true for those marginalized by race, socioeconomic status, or past negative medical experiences, who may lack the confidence to express concerns about hormonal symptoms (29–31). This issue is further exacerbated by the frequent disconnect between healthcare providers and patients seeking contraceptive advice (22, 32, 33). As a result, patients' experiences may be dismissed, leading them to rely on commercialized alternative information sources. The commercialization of PBCS-related products and services further complicates this issue. While these offerings may appeal to those experiencing post-pill symptoms, they also raise concerns about the potential exploitation of vulnerable individuals seeking solutions.

To our best knowledge, there is currently no scientific debate regarding the PBCS which leaves possible affected individuals to the information they find online. This opens the door to doubt, deprivation, and disempowerment, but also subtly perpetuates health inequalities by directing supplement-based “solutions” towards those with the financial means and cultural knowledge to purchase these products and navigate the healthcare system.

## 4 The post-birth control syndrome and (social) media: when biomedicine fails to deliver, who jumps in to fill the gap?

A quick search on German and English language social and media websites using the terms “post-pill syndrome” or “post-birth control syndrome” reveals a large amount of content on what to expect, what happens, and tips for the time after the discontinuation of the contraceptive pill. Building on such a cursory search, we describe the PBCS-related information on web pages found via Google, Instagram, and Reddit. These platforms were selected for their significant role in daily health information searches, peer-to-peer interactions, and wellness content driven by influencers. We included content from English and German language and narratively summarized the data.

### 4.1 Websites

During our online search, we first identified media outlets focusing on medicine and health in general or women's (health) topics (34–38). And second, websites by (women's) health companies (39–42) and webpages by health care specialists, often focusing on Naturopathy, Natural Medicine, or Holistic Women's Health (German: “Ganzheitliche Frauenheilkunde”) (43–51).

All web pages follow a recurring pattern of introducing the PBCS: They define PBCS and explain the underlying mechanism, referring to hormonal “chaos” (42) or “imbalance” (35). This “chaos” is usually explained by the fact that most hormonal birth controls interrupt and suppress the body's natural function of the hormonal system and reproductive processes. With the withdrawal of “exogenous synthetic hormones” (35), the female body relies on natural hormonal production again. This transition can run smoothly, that is, menses occur regularly, and no other bodily symptoms occur. However, this transition may also cause symptoms such as “*Changes in […] menstrual period, like lighter, heavier, or missed periods, mood swings or PMS-like symptoms, Headaches, Acne, Hair loss, Changes in sex drive, Breast tenderness.*” (38). Some web pages provide more in-depth information on the underlying medical issues that may cause PBCS. For instance, Lindsay Schlengel on the web page “Natural Womanhood” interviewed Joana Brighton and writes: “*She says, “the underlying causes of post-birth control syndrome can involve nutrition deficiencies, HPA axis dysregulation [adrenal fatigue], impaired liver detox, stress, and more.” Worse, these problems may not resolve on their own*.” (37).

The web pages often provide health advice and emphasize that women should treat symptoms and help their bodies adjust. Considerations for such support are “*over-the-counter (OTC) pain medications for headaches and menstruation cramps*” (34), “*Staying hydrated, eating green leafy vegetables*” (51), “*restore the nutrients*” (52), or “*your liver might benefit from extra support […] Talk to your provider about targeted herbs and supplements that help support hormone detoxification*.” (50).

Web pages by women's health companies and healthcare providers usually also sell products or consultations related to the PBCS [e.g., supplements (49, 50) or menstruation products (41, 42)].

In addition, web pages by media companies and women's health companies often emphasize that their articles have been medically reviewed (40). In addition, they may refer to PBCS as “*controversial*” (36), “*debate over one term*”, and “*not an official medical diagnosis*” (39), however emphasizing that “*naturopaths say, that doesn't mean it's not real*” (35), or that “some women* report symptoms after stopping the pill” (39). In contrast to that, web pages of those health care professionals who built their careers on PBCS tend to be rather uncritical about this debate and treat PBCS like a medical diagnosis.

### 4.2 Social media – Instagram & Reddit

The #postpillsyndrome has 1.095 and the #postbirthcontrolsyndrome 9.795 posts and reels (June, 3rd 2024). These posts are from medical doctors and self-proclaimed women's health experts, such as “hormonal consultants” and former users who use these platforms to share their experiences.

The content of this debate is similar to the web pages: that the contraceptive pill depletes the body's vitamin reservoirs; how to restore hormonal balance after discontinuation; and what to expect post-discontinuation.

On the social media platform Reddit, individuals in the subreddit r/birthcontrol (53) (“Post birth control syndrome”) take the chance to exchange the problems they encounter after the discontinuation of the pill; they talk about acne, hair loss, less energy, or menstrual problems. Sharing their experiences they write “*i'm glad i'm not alone*”, “*I'd say it took a solid 6–9 months to truly feel like myself.*”, “*I have been dealing with my post birth control syndrome for 2 1/2 years now*” or “*Really considering starting birth control again*”. These stories convey not only physical unease but also a quest for validation and a shared sense of meaning-making (54, 55). This involves users collaborating to interpret symptoms and side effects related to birth control methods, particularly in pseudonymous settings such as Reddit, which allow for more intimate and detailed storytelling. As McDowall et al. (56) demonstrate, such online discussions reflect a unique combination of strategies, including storytelling, risk analysis, and causal reasoning, that help individuals navigate complex and often stigmatized healthcare experiences.

These accounts show the real-life struggles of people coming off birth control. Therefore, it is not surprising that they seek an explanation of their condition. The term “post-pill syndrome” appears to encapsulate these experiences.

## 5 Discussion: why is PBCS a public health issue?

After the brief description of the social media discourse on PBCS, we now want to outline some critical perspectives that highlight the problems of this health data gap from a feminist standpoint (57).

In our cursory research on websites and social media, we found abundant information and media about PBCS. Besides a small segment of conversations between former pill users who use this opportunity to share their experiences, much of the debate around PBCS is dominated by companies and individuals who have economic interests in the topic. From a feminist perspective, these actors filling the void left by biomedicine's indifference to the topic is problematic, as they disseminate misinformation about womxn's health and bodies, as well as recommendations for managing physiological changes following cessation based on commercial interests and largely lack proof of efficacy. In a way, the demedicalization of post-contraception experiences makes space for the further commercialization of womxn's health and their experience of their reproductive agency.

This market is successfully opened through the limited availability of credible information regarding female bodies during and after oral contraceptive pill use. This nonproduction of knowledge creates a power dynamic between former users and the sellers of products and information, while the former users are not empowered to take control of their bodies. While biomedicine and its tendency to medicalize phenomena have been criticized for many decades for a similar power dynamic between patients and health professionals (58), more recent scholarship has highlighted that the medicalization of certain phenomena can also function in the interest of people-turned-patients since biomedicine claiming responsibility often mobilizes resources (59).

The case of PBCS illustrates this argument and highlights a “*systematic failure of scientific thought to account for women as agents and subjects, and for their experiences to be included in determining the definition of problems given attention by science*” (43). However, such systemic neglect does not necessarily lead to womxn stepping up to empower themselves in countering biomedicine's oversights; instead, it might create a void that other disempowering forces can occupy*.*

This connects to a broader issue in public health: Meeting sexual and reproductive justice should focus not only on the prevention of unwanted pregnancies, which is a predominant concern in public health (21, 60). Medical care should be able to support people with credible information regarding all contraceptive choices and hereby pay attention to their experiential lifeworlds, not just to narrowly defined public health targets. This also includes the decision to discontinue, which should also be an informed choice. Contraceptive health research often focuses on the reasons for discontinuation and adherence to contraceptives (61, 62). Therefore, research misses that individuals choose to discontinue the contraceptive pill to put their health and body first (21, 24), or for some other good reason.

However, research that focuses solely on the return of menstruation and fertility is insufficient for making informed decisions. A feminist perspective [or standpoint (57)] calls for research that examines the effects on bodies after discontinuation, beyond the question of reproduction. Moving away from the androcentric paradigm that dominates knowledge production in public health and medicine, a feminist standpoint calls for the reorientation of scientific inquiry. By incorporating the lived experiences and values of womxn, this approach seeks to produce more nuanced, situated, and emancipatory understandings of social phenomena (57, 63). Hence, we need to center research on the (dis)continuation of contraceptives around womxn's needs. Such information would not only enable (former) pill users to take charge of their health and bodies, but it would also promote reproductive justice in medical and public health research and practice and empower womxn to make informed decisions when choosing health-related products and information.

Research should emphasize this and support individuals in their decisions by providing evidence that can be used in contraceptive counseling. Therefore, we call for classical clinical and epidemiological research, exploring the underlying mechanisms of symptoms that former pill users may experience post-discontinuation. In addition, we emphasize the need for qualitative research to explore individual experiences and needs that incorporate sexual and reproductive health rights and a rights-based epistemology.

## 6 Conclusions

This perspective highlights the critical need to address the systemic knowledge gap regarding the effects of discontinuing OCPs, particularly through a feminist lens. Current discourse on PBCS often lacks credible information, leaving former pill users vulnerable to misinformation and commercial exploitation. We contend the importance of reframing contraceptive health research in prioritizing individuals' (post-dis)continuation experiences and needs. Instead of focusing merely on risk prevention of a potential pregnancy, focus should shift to supporting the sexual and reproductive agency and choices. Merely addressing this knowledge gap is insufficient. Social epidemiological studies have shown that medical and contraceptive information is unevenly distributed and is influenced by social factors such as race, socioeconomic status, education, and healthcare system interactions. To improve equity in sexual and reproductive health, we must examine how knowledge is generated, shared, and accessed. This involves community participation in research, customizing information for varied audiences, and ensuring that clinical advice is culturally appropriate, inclusive, accessible, and not just theoretical.

Such efforts would empower individuals with the knowledge required for informed decision-making and reshape public health and medical practices to better align with the principles of sexual and reproductive health rights. Although clinical and social epidemiological research is needed to better understand the mechanisms underlying contraceptive pill discontinuation, it is important to consider the context in which such interventions are implemented. Attention to these contexts can support a more effective transfer and adaptation of interventions across different settings. They should be viewed as events occurring within complex systems rather than simple technical procedures that can be applied uniformly across contexts (64).

## Data Availability

The original contributions presented in the study are included in the article/Supplementary Material, further inquiries can be directed to the corresponding author.

## Footnotes

^1^In our opinion, all people regardless of their gender should have access to sexual and reproductive health rights. Therefore, we use the term womxn to include all people regardless of their gender. We will only use the term women in this perspective, if the authors of the original reference use it. We do this intentionally to emphasize the gendered heteronormative discussion in our references.

^2^Feminist research perspectives not only prioritize women's experiences and voices (65), they acknowledge that research is shaped by the existing power dynamics and social contexts (5). In addition, research using a feminist methodology calls for action, emphasizing engagement, empowerment, reflexibility, and social change (66).
